# Plasma organochlorine concentrations and bone ultrasound measurements: a cross-sectional study in peri-and postmenopausal Inuit women from Greenland

**DOI:** 10.1186/1476-069X-5-33

**Published:** 2006-12-21

**Authors:** Suzanne Côté, Pierre Ayotte, Sylvie Dodin, Claudine Blanchet, Gert Mulvad, Henning S Petersen, Suzanne Gingras, Éric Dewailly

**Affiliations:** 1 Unité de recherche en Santé publique, Centre de recherche du CHUL-CHUQ, Québec, QC, G1V 5B3, Canada; 2 Département de Médecine Sociale et Préventive, Université Laval, Québec, QC, G1K 7P4, Canada; 3 Centre Ménopause Québec, Hôpital St-François D'Assise (CHUQ), Québec, QC, G1L 2G1, Canada; 4 Unité de Recherche en Endocrinologie de la Reproduction, Centre de Recherche de l'Hôpital St-François D'Assise-CHUQ, Québec, G1L 3L5, Canada; 5 Center for Arctic Environmental Medicine, PO Box 1001 DK-3900, Nuuk, Greenland

## Abstract

**Background:**

Inuit women are highly exposed through their traditional seafood based diet to organochlorine compounds, some of them displaying endocrine disrupting properties. We hypothesized that this exposure might be related to bone characteristics that are altered in osteoporosis, because hormone deficiency is a known risk factor for the disease.

**Methods:**

We measured quantitative ultrasound parameters (QUS) at the right calcaneum of 153 peri- and postmenopausal Inuit women (49–64 year old) from Nuuk, Greenland, and investigated the relation between these parameters and plasma organochlorine concentrations. We used high-resolution gas chromatography with electron capture detection to analyze plasma samples for 14 polychlorinated biphenyls (PCB) congeners and 11 chlorinated pesticides and metabolites. We analysed morning urine samples for cadmium, a potential confounder, by atomic absorption spectrometry. We used a validated questionnaire to document dietary and lifestyle habits as well as reproductive and medical histories.

**Results:**

Concentrations of PCB 153, a surrogate of exposure to most organochlorines present in plasma samples, were inversely correlated to QUS parameters in univariate analyses (*p *< 0.001). However, PCB 153 concentrations were not associated with QUS values in multivariate analyses that comprised potential confounding factors such as age, body weight, former oral contraceptive use and current hormone replacement therapy (HRT) use, which were all significant predictors of bone stiffness (total R^2 ^= 0.39; *p *< 0.001).

**Conclusion:**

Overall we found little evidence that organochlorines exposure is related to osteoporosis in Greenlandic Inuit women, but the hypothesis that exposure to dioxin-like compounds might be linked to decreased bone quality and osteoporosis deserves further attention.

## Background

Osteoporosis is commonly defined as a decrease in bone mineral density (BMD) and the microarchitectural deterioration of bone tissue [1]. It is a multifactorial chronic disease that may progress silently for decades until characteristic fractures occur late in life. This bone fragility increases considerably the risk of osteoporotic fractures, such as those of the distal radius, humerus, spine and hip that often appear in older women following a minimal trauma. Hip fractures lead to rehabilitation problems and greatly decrease the quality of life [2,3].

Risk factors for osteoporosis in women include advanced age, small body size, cigarette smoking, hormone deficiency, genetics, low physical activity, low intake of calcium and vitamin D, menopausal status, the use of certain drugs (eg, glucocorticoids) as well as several medical disorders [4,5]. Exposure to environmental chemicals that are able to disrupt the hormonal equilibrium might represent another risk factor for this disease [6]. More specifically, in view of the important role of estrogen deficiency in the osteoporotic process [7], compounds that can interact with estrogen receptors or alter estrogen metabolism could be involved in the pathogenesis. Certain environmental chemicals that are part of the organochlorine (OC) family, a group of persistent and bioaccumulative chemicals that have been used extensively in agriculture and various industrial applications between 1930 and 1980, can modulate the estrogen signalling pathway, namely polychlorinated biphenyls [8], β-hexachlorocyclohexane [9] and 2,3,7,8-tetrachlorodibenzo-*p*-dioxin and structurally-related compounds [10-12]. Results from two recent studies suggest a possible relation between osteoporosis and exposure to OCs [13,14].

High concentrations of OCs have been found in plasma samples from adult Greenlanders compared to those reported for southern Canada and European populations [15,16]. OCs are transported from industrialized areas of the planet to the Arctic by ocean and air currents [17-19], where they condensate and are biomagnified in predator species located at the top of the marine food chain, such as predator fish and sea mammals [17,18,20]. The traditional Inuit diet comprises large amounts of sea mammal and is the major source of OC exposure in this population [17,18,20-22]. In view of the unusually high exposure to OCs in Greenland, we set out to examine the associations between OCs plasma concentrations and ultrasound bone measurements among peri- and postmenopausal Greenlandic Inuit women. QUS parameters have been shown to be strongly associated with future fracture risk [23,24]. We also measured the concentration of cadmium in urine because low-level cadmium exposure has been linked to osteoporosis [25,26] and therefore it represents a possible confounding factor.

## Methods

### Population

This descriptive cross sectional study was conducted during September 2000 in Nuuk, Greenland, home to a total population of approximately 14,000 inhabitants. To be eligible, women had to be born in Greenland and aged between 49 and 64 years. A random sample of 200 women was taken from the Greenland statistics list comprising 547 eligible women. Of the 200 women, 7 had died, 11 had moved to another town and 15 were out of town at the time of the study (not for medical reasons). Thus, 167 women were invited to participate to the study; eight declined, for a participation rate of 95%. Six women were excluded because of illnesses (human immunodeficiency virus, mental disorder and flu) and therefore 153 women completed the study.

### Measurements and analyses

After obtaining information on the study and signing an informed consent, participants completed a detailed and validated Danish questionnaire with the assistance of a qualified interviewer during a face-to-face interview. The questionnaire was adapted from the Mediterranean Osteoporosis Study Questionnaire (MEDOS) [27] and allowed to document the following risk factors for osteoporosis: smoking habits, physical activity, daily milk products and calcium supplement consumption, current use of HRT and former contraceptive use. Questions were also asked to document secondary causes of osteoporosis such as Cushing's disease, renal and liver deficiency, hyper- and hypothyroidism, bone cancer and rheumatoid arthritis.

Weight, height and waist, abdominal and hip girth were measured using standardized techniques. Women were considered postmenopausal if they had no menses for at least one year before the recruitment, if they underwent bilateral oophorectomy more than 6 months ago and if analysis had revealed a follicle stimulating hormone concentration greater than 40 IU/L.

Blood samples (10 ml) were collected in vials containing EDTA as the anticoagulant, centrifuged (10 min, 3000 rpm) and the plasma poured into glass vials pre-rinsed with hexane. First morning urine samples were collected in plastic vials. Plasma and urine samples were stored frozen at -80°C until time of analysis for OCs and cadmium respectively, at the *Centre de Toxicologie *(CTQ) of the *Institut National de Santé Publique du Québec *(Québec, Canada). This laboratory is accredited by the Canadian Association for Environmental Analytical Laboratories.

Fourteen PCB congeners [International Union for Pure and Applied Chemistry (IUPAC) no. 28, 52, 99, 101, 105, 118, 128, 138, 153, 156, 170, 180, 183, 187], *p*, *p*'-DDT (dichlorodiphenyltrichloroethane) and its major metabolite *p*, *p*'-DDE (*p*, *p*'-dichlorodiphenyl-dichloroethylene), hexachlorobenzene (HCB) and 8 other chlorinated pesticides [α-chlordane, γ-chlordane, aldrin, β-hexachlorocyclohexane (β-HCH), mirex, oxychlordane, *cis*-nonachlor, and *trans*-nonachlor] were analysed by high-resolution gas chromatography with electron capture detection. A 1:1:3 mixture of ammonium sulfate:ethanol:hexane was first added to the plasma to extract OCs. The extracts were then concentrated and purified on two Florosil columns (60–100 mesh; Fisher Scientific, Nepean, Ontario, Canada). The OCs were measured in the purified extracts with an HP 5890 high-resolution gas chromatograph equipped with dual-capillary columns (HP Ultra I and Ultra II) and dual Ni-63 electron capture detectors (Hewlett-Packard, Palo Alto, CA, USA). The limit of detection was based on 3 times the average standard deviation of noise and was 0.08 μg/L for *p*, *p*'-DDE, *p*, *p*'-DDT and β-HCH, and 0.04 μg/L for all other compounds. The average percent recoveries were greater than 95%. Coefficients of variations (CVs) based on repeated analyses of a standard reference material (SRM 1589; N = 15) ranged from 3.9% to 18.5%, except for PCB 105 and *p*, *p*'-DDT, for which CVs were 31.6% and 26.2%, respectively.

Plasma OC concentrations were expressed on a lipid basis. We calculated the total plasma lipid concentration from the concentrations of cholesterol esters, free cholesterol, triglycerides and phospholipids [28], which were measured using standard enzymatic procedures.

Urine samples were analysed for cadmium by graphite furnace atomic absorption spectrometry and concentrations were corrected for urinary creatinine content to take into account differences in urinary output between participants. Duplicates were run every 10 samples. The CV of the method based on repeated analyses of a standard reference material from CTQ interlaboratory comparison program (N = 34) was 2.0%, and the limit of detection was 0.2 μg/L.

### Bone measurements

QUS bone measurements were performed at the right calcaneum using the Achilles™ ultrasound bone densitometer (Lunar Corporation, Madison, WI, USA). This technique is fast (approximately 3 minutes), simple, non invasive, safe (radiation-free), inexpensive and portable. The heel is immersed in water and an ultrasonic pulse propagates through the bone. Three QUS parameters were measured: 1) broadband ultrasound attenuation (BUA, dB/MHz), which reflects bone density as well as its architecture; 2) speed of sound (SOS, m/sec), which reflects bone density and elasticity; and 3) bone stiffness index (SI, %), which reflects the rigidity of the bone structure. SI was computed from BUA and SOS measurements using the manufacturer's equation and expressed as a percentage of young adults' average peak SI. The densitometer was calibrated daily using the acoustic phantom provided by the manufacturer and showed no drift. *In vivo *precision was evaluated by repeated measurements conducted among 15 subjects: CVs were 0.8% for BUA, 0.2% for SOS and 1.1% for SI.

### Statistical analysis

Because distribution of values for organochlorine concentrations in plasma lipids and urinary cadmium concentrations were skewed right, they were log-transformed and the geometric mean was used as the measure of central tendency. A concentration equal to half of the detection limit was given for samples with levels below the detection limit. Means of bone measurements were compared for dichotomous risk factors using Student's *t *tests. We used Pearson's correlation coefficients to assess correlations between QUS measurements and continuous risk factors. PCB 153 was used as a surrogate of the organochlorine mixture present in the traditional diet because concentrations of this congener in plasma were strongly correlated to those of most other OCs. Nevertheless, since β-HCH, *p*, *p*'-DDE and HCB were not as strongly correlated to PCB 153 (Pearson's r = 0.75–0.78), statistical analyses were also performed with these variables. In order to verify the association between bone measurements and concentrations of OCs, multivariate regression analyses were performed. We evaluated the confounding effect of osteoporosis risk factors on the relation between OCs and QUS parameters and retained only factors associated with bone measurements (*p *≤ 0.10) in the regression models. Statistical significance was set at α = 0.05. Multiple linear regression assumptions regarding the homogeneity of variance and the normality of residues were met when log-transformed OC concentrations were entered in the models. Statistical analyses were performed using SAS Statistical program version 8.0 (SAS Institute Inc., Cary, NC, USA).

## Results

Our study population consisted of 153 Inuit women, mostly postmenopausal whose age ranged between 49 and 64 years (Table 1). Approximately half of the women had a body mass index (BMI) exceeding 27 kg/m^2^. Most were sedentary, smoked cigarettes, and were not taking calcium supplement or HRT at the time of the study. A majority of women also never used contraceptives. Mean (geometric) urinary cadmium concentration was 1.4 μg/g creatinine (95%-confidence interval = 1.3–1.6), with values ranging from 0.2 to 7.6 μg/g creatinine (N = 141).

**Table 1:** Characteristics of the 153 peri- and postmenopausal Inuit women

**Variables**	**Mean ± SD **	**Min**	**Max**	**Variables**	**Categories**	**%**
Age (years)	55.3 ± 4.4	49.0	64.0	Smoking habits	Smoker Former smoker Never smoker	74.5 11.8 13.7
Weight (kg)	68.0 ± 15.2	36.0	102.0	Physical activity	Sedentary Active*	73.9 26.1
Height (cm)	156.2 ± 6.6	142.0	171.0	Dairy products (Portions per day)	0 to 2 3 and more	71.3 28.7
Body mass index (kg/m^2^)	27.9 ± 6.1	14.4	43.4	Calcium supplement use	Yes No	8.5 91.5
Waist circumference (cm)	93.4 ± 14.4	64.0	131.0	Menopausal (n = 141)	Yes No	87.9 12.1
Abdominal circumference (cm)	96.3 ± 14.2	67.0	129.0	Current HRT use	Yes No	8.5 94.8
Hip circumference (cm)	103.3 ± 10.3	82.0	130.0	Former contraceptive use	Yes No	40.5 59.5

*Active: at least one period of physical activity (20–30 min duration) per week over the last three months; sedentary: less than one period of physical activity per week.

Concentrations of the 25 OCs measured in plasma samples from the 153 participating women are shown in Table 2. Data are provided only for compounds for which 70% of the samples contained concentrations above the detection limit of the analytical method. The geometric mean concentration of the sum of 14 PCB congeners (ΣPCBs) was 2051 μg/kg. The most prominent congeners were PCB 153, PCB 138 and PCB 180. These three di-ortho substituted congeners represented 66% of ΣPCBs. Three mono-ortho substituted congeners – PCB 105, PCB 118 and PCB 156 – were detected in almost all samples and represented 11% of ΣPCBs. *p*, *p*'-DDE, the main DDT metabolite, was the chlorinated pesticide with the highest plasma concentration (mean concentration above 1000 μg/Kg), followed by *trans*-nonachlor, hexachlorobenzene, and oxychlordane, with mean concentrations between 200 and 400 μg/Kg.

**Table 2:** Plasma organochlorine concentrations (μg/kg lipids) in the 153 peri- and postmenopausal Inuit women

**Variables**	**% detected**	**Geometric mean (95% CI)**	**Min**	**Max**
**PCBs (IUPAC No)**				
28	83	7.7 (6.8–8.7)	ND*	111.1
52	76	7.3 (6.4–8.4)	ND	80.0
99	100	88.0 (80.1–96.6)	9.3	294.7
101	84	8.9 (7.9–10.0)	ND	36.6
105	98	22.5 (20.2–25.1)	ND	77.7
118	100	121.9 (111.9–132.8)	19.7	371.8
128	23			
138	100	418.2 (384.4–454.9)	70.6	1385
153	100	578.6 (530.2–631.3)	93.8	1993
156	100	77.2 (70.9–83.9)	11.4	295.9
170	100	132.5 (121.4–144.6)	20.7	606.4
180	100	350.6 (319.8–384.4)	57.9	1709
183	100	41.7 (38.3–45.4)	7.4	160.0
187	100	164.3 (150.7–179.0)	24.2	660.3
ΣPCBs	100	2051 (1886–2229)	341	7384
**Chlorinated pesticides**				
Aldrin	3			
β-HCH	100	49.4 (46.3–52.6)	16.2	134.6
γ-Chlordane	25			
*cis*-Nonachlor	100	87.2 (79.3–96.0)	9.5	287.5
*p*, *p*'-DDE	100	1192 (1083–1313)	94.2	6464
*p*, *p*'-DDT	95	33.5 (29.9–37.6)	ND	145.6
HCB	100	289.0 (264.3–316.0)	56.9	1458
Mirex	100	39.6 (34.9–45.0)	ND	242.4
Oxychlordane	100	214.6 (192.6–239.0)	15.9	1268
*trans*-Nonachlor	100	388.7 (351.8–429.5)	40.7	1903

*ND; not detected

**Figure 1:**
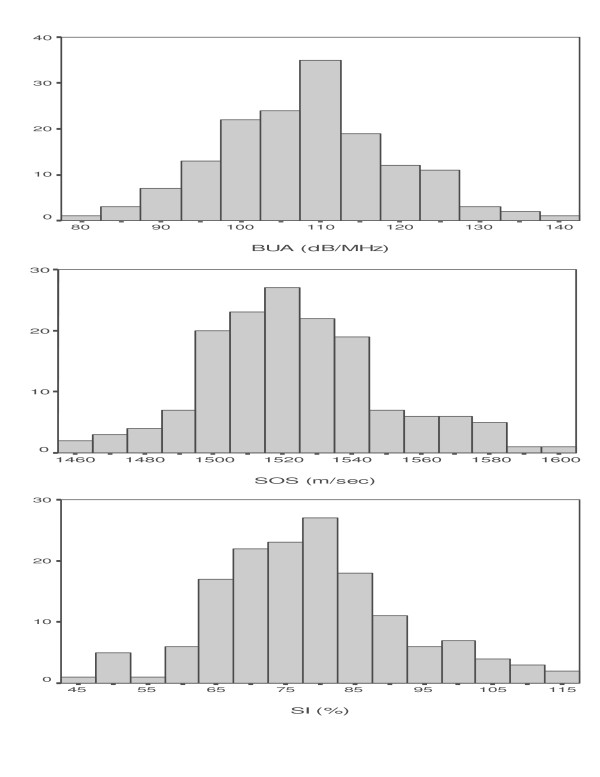
Frequency distributions of right calcaneal QUS bone measurements in 153 peri- and postmenopausal Inuit women.

Frequency distributions for QUS parameters measured at the right calcaneum of the 153 women are shown in Figure 1. Mean values for BUA, SOS, and SI were respectively 108 dB/MHz (Standard deviation (SD) = 11; range = 68–140), 1523 m/sec, (SD = 26; range = 1463–1595) and 79% (SD = 14; range = 47–117).

We first tested relations between QUS parameters and selected dichotomous risk factors for osteoporosis and noted that postmenopausal women, women who were not currently using HRT and those who never used contraceptives had significantly lower values of all three QUS parameters compared to their respective counterparts (Table 3). The level of physical activity was marginally linked to BUA, SOS and SI values. Smoking and taking calcium supplements only influenced BUA values, while the consumption of dairy products had no statistically-significant influence on QUS measurements. Correlation analyses revealed that age was negatively correlated to all three QUS parameters (Pearson's r ranging from -0.31 to -0.42; *p *< 0.001). All anthropometric measurements were positively correlated to QUS measurements but the strongest correlations were observed for weight (r = 0.33 – 0.50; *p *< 0.001) and height (r = 0.41 – 0.46; *p *< 0.001). Urinary cadmium concentrations were negatively correlated to QUS parameters (r varying from -0.23 to -0.29; *p *< 0.005).

**Table 3:** Univariate analyses of relations between quantitative ultrasound bone measurements and risk factors for osteoporosis (n = 153)

	**BUA (dB/MHz)**		**SOS (m/sec)**		**Stiffness index (%)**	
	Mean **± SD**	*p*-value*	Mean **± SD**	*p*-value*	Mean **± SD**	*p*-value*

Smoking habits						
Smoker	107.2 ± 11.5	0.025	1521.9 ± 27.3	0.409	77.5 ± 14.5	0.099
Former and never smoker	110.9 ± 7.7		1525.9 ± 22.4		81.1 ± 10.5	
Physical activity						
Sedentary	107.2 ± 10.9	0.075	1520.6 ± 25.2	0.064	77.2 ± 13.4	0.048
Active	110.8 ± 10.0		1529.5 ± 28.0		82.1 ± 13.7	
Dairy products						
0 to 2/day	108.4 ± 10.8	0.668	1523.3 ± 26.6	0.767	78.7 ± 13.7	0.713
≥3/day	107.6 ± 10.8		1521.9 ± 25.2		77.8 ± 13.5	
Calcium supplement						
Yes	114.2 ± 5.6	0.001	1527.8 ± 22.0	0.485	83.9 ± 9.2	0.130
No	107.6 ± 11.0		1522.5 ± 26.5		78.0 ± 13.9	
Menopause						
Yes	106.7 ± 9.9	0.024	1518.9 ± 24.7	0.001	76.3 ± 12.6	0.003
No	112.8 ± 13.5		1540.4 ± 28.9		86.5 ± 16.3	
Current HRT use						
Yes	117.1 ± 9.7	0.002	1546.9 ± 20.0	< 0.001	91.3 ± 10.8	< 0.001
No	107.3 ± 10.5		1520.7 ± 25.6		77.3 ± 13.2	
Former contraceptive use						
Yes	112.1 ± 9.8	< 0.001	1534.3 ± 25.1	< 0.001	84.3 ± 12.7	< 0.001
No	105.4 ± 10.6		1515.1 ± 24.0		74.5 ± 12.8	

*Student *t *Test

Correlations coefficients between QUS parameters and concentrations of selected organochlorines in plasma are listed in Table 4. Concentrations of PCB 153 (log-transformed) were negatively correlated to BUA, SOS and SI measurements (*p *< 0.001). We also tested correlations between QUS parameters and other OCs that were not strongly correlated to PCB 153 levels. Hexachlorobenzene concentrations were correlated with the three QUS parameters, but not those of *p*, *p*'-DDE and β-HCH.

**Table 4:** Pearson's correlation coefficients (r) between quantitative ultrasound bone measurements and plasma organochlorines concentrations (n = 153)

	**BUA (dB/MHz)**		**SOS (m/sec)**		**Stiffness index (%)**	
	*r*	*p*-value	*r*	*p*-value	*r*	*p*-value

PCB 153 (log)	-0.29	< 0.001	-0.32	< 0.001	-0.33	< 0.001
β-HCH (log)	-0.02	0.784	-0.09	0.252	-0.06	0.427
*p*, *p*'-DDE (log)	-0.06	0.479	-0.10	0.236	-0.08	0.306
HCB (log)	-0.17	0.035	-0.26	0.001	-0.23	0.004

Results of multiple linear regression analyses are presented in Table 5. PCB 153 was not associated with QUS parameters in models that included potential confounding factors, although associations were nearly significant in SOS and BUA models (*p *= 0.072). The significant independent predictors of QUS measurements were age, weight, current HRT use and former oral contraceptives use. These variables accounted for 38%, 34% and 39% of the variance in BUA, SOS and SI values respectively (*p *< 0.001). Urinary Cd concentration was not retained in the final regression models as it was not associated with any of the bone measurements (p > 0.7). HCB concentrations were also not associated with QUS parameters in multivariate analyses (data not shown). Additional exploratory analyses were conducted with concentrations of mono-ortho substituted PCB congeners, even though concentrations of these congeners were highly correlated to those of PCB 153 (Pearson's r ≥ 0.90). In similar models (data not shown), statistically significant associations were noted between PCB 156 concentrations and BUA (β = -8.12; standard error (SE) = 3.65; *p *= 0.028), SOS (β = -22.68; SE = 9.07; *p *= 0.014) and SI values (β = -11.95; SE = 4.53; *p *= 0.009). Concentrations of PCB 105 and PCB 118 were not associated with QUS parameters in multivariate models.

**Table 5:** Multiple regression models for predicting quantitative ultrasound bone measurements (n = 153)

**Variables**	**BUA (dB/MHz)**		**SOS (m/sec)**		**Stiffness index**	
	β (SE)*	*p-value*	β (SE)*	*p-value*	β (SE)*	*p-value*

R^2^	0.38	<0.001	0.34	<0.001	0.39	<0.001
PCB 153 (log)*	-4.59 (3.20)	0.153	-14.41 (7.95)	0.072	-7.19 (3.98)	0.072
Age (years)	-0.33 (0.18)	0.071	-1.54 (0.45)	0.001	-0.65 (0.22)	0.004
Weight (kg)	0.31 (0.05)	<0.001	0.40 (0.12)	0.001	0.31 (0.06)	<0.001
Current HRT use	6.26 (2.60)	0.017	16.31 (6.46)	0.013	8.93 (3.23)	0.006
Former contraceptive use	3.38 (1.56)	0.031	9.78 (3.87)	0.012	4.93 (1.94)	0.012

*Regression coefficients (standard error)

## Discussion

This cross-sectional study was designed to investigate the association between exposure to OCs and osteoporosis-related ultrasound bone measurements in peri- and postmenopausal Inuit women from Greenland. Exposure of the Greenlandic Inuit population to OCs is high compared to North American and European populations [15] and we hypothesized that exposure to these compounds might favor the osteoporotic process. We found no statistically-significant association between QUS parameters and concentrations of PCB 153, a surrogate for exposure to the majority of OCs present in plasma samples of participants.

Some studies have investigated the relation between biomarkers of OC exposure and the risk of fractures in women. Alveblom et al. [13] compared fracture risks between fishermen and their wives from the Swedish east coast on the Baltic Sea (exposed to OCs through fatty fish consumption) and west coasts (unexposed). The authors retrieved information on vital status and hospitalization of 17,823 persons from 1987 to 1996, among which 671 had been hospitalized due to osteoporotic fractures. Poisson regression models taking age and calendar year into account revealed a significantly increased incidence rate ratio (IRR: 2.29, 95% confidence intervals (CI): 1.23–4.28) for vertebral fractures among east-coast women. The authors could not exclude that confounding from differences in smoking habits between the populations might explain part of the observed effects. Wallin et al [29] conducted a questionnaire study to further assess the impact of consuming OC-contaminated fish on the self-reported fracture incidence in fishermen and their wives from Swedish east and west coasts. Hip, vertebral, and wrist fractures were classified as osteoporotic. No differences in fracture incidence were observed between the east-coast (exposed) and west-coast (unexposed) cohorts. East-coast wives with more than one meal of fatty fish from the Baltic Sea per month had, however, an increased fracture incidence as compared with that of the east-coast wives who ate, at most, one such a meal per month (age-adjusted IRR = 1.68, 95% CI = 1.00–2.84). In both of these studies, OC exposure was indirectly assessed through fish consumption, which might have introduced misclassification of exposure.

Few studies investigated relations between OC exposure and bone mineral density measurements. No association was found between plasma *p*, *p*'-DDE concentrations and BMD measured at the lumbar spine and radius in 103 peri- and postmenopausal American women (mean age = 54.5 years) who had participated in the Mount Sinai Medical Center Longitudinal Normative Bone Density Study from 1984 to 1987 [30]. Beard et al. [14] examined the relationship between serum *p*, *p*'-DDE concentrations and bone mineral density in 68 Australian sedentary women aged 45 to 64 years old. The authors observed that *p*, *p*'-DDE levels (mean serum concentration = 3.9 ppb) were negatively correlated to BMD (r = -0.27, *p *= 0.03). The strongest multivariate model explained 21% of bone mineral density variation (*p *= 0.002) and comprised log-transformed *p*, *p*'-DDE concentrations (*p *= 0.018), age (*p *= 0.002), and years of HRT use (*p *= 0.10) as predictor variables. Finally, Wallin et al. [31] recruited 184 wives of fishermen from the east coast of Sweden (median age 62 years) to participate in an examination of their forearm BMD, using dual energy x-ray absorptiometry (DXA). Univariate analyses showed significant negative associations between BMD and PCB 153 or *p*, *p*'-DDE concentrations, but after adjustment for age and body mass index, these associations did not remain, similarly to what was observed in the present study.

Additional exploratory analyses in our study revealed significant associations between QUS bone measurements and PCB 156 concentrations, a congener with dioxin-like properties. Animal studies have shown that dioxin-like OCs may impair normal bone metabolism and result in increased bone fragility [32-34]. PCB 126, a non-ortho coplanar PCB congener, impaired the mineralization process of tibiae in rats [33] and also reduced the collagen content and serum osteocalcin concentrations, resulting in impaired maximum torque and stiffness of the rat humerus [34]. Although biologically plausible, the association noted between PCB 156 and QUS parameters may be due to chance and needs to be replicated in another study in which all dioxin-like OCs would be measured and taken into consideration in statistical analyses.

Data on bone measurements and osteoporosis risk factors obtained in the 153 Inuit women can be compared to those gathered from 2972 southern Quebec women of the same age group (mean = 55.8 years, ranging form 49–64 years old) who were recruited during the same period by our research group (unpublished data). The same questionnaires and ultrasound apparatus were used for data collection in both settings. The mean ± SD for BUA in southern Quebec women was 112 ± 9 dB/MHz, 1545 ± 29 m/sec for SOS and 87 ± 13% for SI. Mean values for BUA, SOS and SI in Inuit women from Greenland are respectively 4%, 1.4% and 9.5% lower that those measured in southern Quebec women. A larger proportion of Inuit women smoked (74.5% vs 14.5%) and was sedentary (73.9% vs 24.7%), while a smaller proportion of Inuit women was taking calcium supplements (8.5% vs 46.7%) and HRT (8.5% vs 51.9%) compared to southern Quebec women. Furthermore, mean weight (68.0 vs 66.4 kg) and BMI (27.9 vs 26.5 kg/m^2^) were slightly higher in Greenlandic Inuit women than in southern Quebec women. These differences in osteoporosis risk factors might explain the lower values for QUS measurements obtained in Greenlandic Inuit women compared to women from southern Quebec.

Other studies conducted in Alaska have shown that Inuit women are at risk for osteoporosis. A study conducted in the 70's using direct photon absorptiometry revealed that Alaskan Inuit women had an earlier onset and greater intensity of bone loss beginning in the forties, around menopause, compared to Caucasian women in the US. Alaskan Inuit women lost on average 5% more bone mass per decade than Caucasian American women [35,36]. Martin et al. [37] reported that the bone mineral content of Inuit women decreased by 50% between the third and sixth decades. More recently, Filner et al. [38] evaluated the prevalence of risk factors for osteoporosis in women from 13 geographic areas in Alaska and noted that low bone density (defined as a T-score of -1.0 or less) measured at the calcaneum by ultrasonography was more prevalent in Native than non-Native women (45.3% in Native vs 22.1% in non-Native, *p *< 0.001). These authors concluded that increasing calcium intake and decreasing the prevalence of smoking would likely reduce the prevalence of osteoporosis in Native women.

A limitation of our study is its cross-sectional design, which can lead to an underestimation of the true association because some subjects might not have been available at the time of ascertainment. Moreover, the exposure is measured at the same time as the health condition of the subject, which makes it difficult to correctly establish a causal relation. Also, our assessment of the physical activity was based on a single question, which documented how many times per week during the last three months had the woman practiced a physical activity for at least 20 to 30 minutes. This question which was validated in a group of workers from southern Canada [39] might have introduced some information bias. Finally, our study relied on QUS measurements at the right calcaneum to assess bone properties instead of the gold-standard vertebral BMD measurements. Several studies have however shown that QUS measurements are good predictors of the bone density and quality that are related to the risk of osteoporosis [24,40-43] and to the risk of fracture in early postmenopausal women [41,43].

## Conclusion

In summary, we observed that plasma OC concentrations were not associated with QUS parameters in multivariate models adjusted for age, body size and hormone intake. Exploratory analyses indicated that PCB 156, a congener with limited dioxin-like activity, might be related to bone measurements in this population, but this hypothesis clearly needs to be tested in another setting. We are currently studying the relation between OC exposure and QUS measurements in Inuit women from Nunavik (Arctic Quebec, Canada). In this study, the concentration of all dioxin-like compounds in plasma of participants will be measured by the dioxin-receptor chemical-activated luciferase expression (DR-CALUX) assay. Women will be followed prospectively in order to accurately define the onset and the rate of bone loss. In the meantime, actions should be taken to reduce the prevalence of smoking in Greenlandic Inuit women, which is a known risk factor of osteoporosis. In addition, dietary supplementation with calcium and vitamin D [44] might also be warranted in this population.

## Competing interests

The author(s) declare that they have no competing interests.

## Authors' contributions

SC participated in the design of the study, acquisition of data, interpretation of the data, and drafted the manuscript. PA participated in the interpretation of the statistical analysis, helped to draft the manuscript and revised it critically. SD conceived of the study, and helped to draft the manuscript. CB participated in the design of the study, acquisition of data, and helped to draft the manuscript. GM conceived of the study, participated in its design and coordination and helped to draft the manuscript. HSP conceived of the study, participated in its design and coordination and helped to draft the manuscript. SG performed the statistical analysis and helped to draft the manuscript. ED conceived of the study, helped to draft the manuscript, and revised it critically.

## References

[B1] Genant HK, Cooper C, Poor G, Reid I, Ehrlich G, Kanis J, Nordin BE, Barrett-Connor E, Black D, Bonjour LP, Dawson-Hughes B, Delmas PD, Dequeker J, Ragi Eis S, Gennary C, Johnell O, Johnston CC, Lau EM, Liberman UA, Lindsay R, Martin TJ, Masri B, Mautalen CA, Meunier PJ, Khaltaev N (1999). Interim report and recommendations of the World Health Organization Task-Force for Osteoporosis. Osteoporos Int.

[B2] Brantus JF, Delmas PD (1997). Ostéoporose. Épidémiologie, étiologie, diagnostic, prévention. Pathologie de l'appareil locomoteur. Rev Prat.

[B3] Papadimitropoulos EA, Coyte PC, Josse RG, Greenwood CE (1997). Current and projected rates of hip fracture in Canada. CMAJ.

[B4] Lane NE (2006). Epidemiology, etiology, and diagnosis of osteoporosis. Am J Obstet Gynecol.

[B5] North American Menopause Society (2006). Management of osteoporosis in postmenopausal women: 2006 position statement of The North American Menopause Society. Menopause.

[B6] Holmes P, Rumsby P, Harrison PT (2004). Endocrine disrupters and menopausal health. J Br Menopause Soc.

[B7] Deroo BJ, Korach KS (2006). Estrogen receptors and human disease. J Clin Invest.

[B8] DeCastro BR, Korrick SA, Spengler JD, Soto AM (2006). Estrogenic activity of polychlorinated biphenyls present in human tissue and the environment. Environ Sci Technol.

[B9] Steinmetz R, Young PC, Caperell-Grant A, Gize EA, Madhukar BV, Ben-Jonathan N, Biqsby RM (1996). Novel estrogenic action of the pesticide residue beta-hexachlorocyclohexane in human breast cancer cells. Cancer Res.

[B10] Abdelrahim M, Ariazi E, Kim K, Khan S, Barhoumi R, Burghardt R, Liu S, Hill D, Finnell R, Wlodarczyk B, Jordan VC, Safe S (2006). 3-Methyl-cholanthrene and other aryl hydrocarbon receptor agonists directly activate estrogen receptor alpha. Cancer Res.

[B11] Kharat I, Saatcioglu F (1996). Antiestrogenic effects of 2,3,7,8-tetrachlorodibenzo-p-dioxin are mediated by direct transcriptional interference with the liganded estrogen receptor. Cross-talk between aryl hydrocarbon- and estrogen-mediated signaling. J Biol Chem.

[B12] Krishnan V, Safe S (1993). Polychlorinated biphenyls (PCBs), dibenzo-p-dioxins (PCDDs), and dibenzofurans (PCDFs) as antiestrogens in MCF-7 human breast cancer cells: quantitative structure-activity relationships. Toxicol Appl Pharmacol.

[B13] Alveblom AK, Rylander L, Johnell O, Hagmar L (2003). Incidence of hospitalized osteoporotic fractures in cohorts with high dietary intake of persistent organochlorine compounds. Int Arch Occup Environ Health.

[B14] Beard J, Marshall S, Jong K, Newton R, Tripplett-McBride T, Humphries B, Bronks R (2000). 1,1,1-trichloro-2,2-bis (p-chlorophenyl)-ethane (DDT) and reduced bone mineral density. Arch Environ Health.

[B15] Bjerregaard P, Dewailly E, Ayotte P, Pars T, Ferron L, Mulvad G (2001). Exposure of Inuit in Greenland to organochlorines through the marine diet. J Toxicol Environ Health A.

[B16] Deutch B, Pedersen HS, Hansen JC (2004). Dietary composition in Greenland 2000, plasma fatty acids and persistent organic pollutants. Sci Total Environ.

[B17] Arctic Monitoring Assessment Programme (1998). Arctic Monitoring and Assessment Programme: Arctic Pollution Issues. Norway.

[B18] Arctic Monitoring Assessment Programme (2002). Arctic Monitoring and Assessment Programme: Human Health in the Arctic. Norway.

[B19] Hung H, Blanchard P, Halsall CJ, Bidleman TF, Stern GA, Fellin P, Muir DC, Barrie LA, Jantunen LM, Helm PA, Ma J, Konoplev A (2005). Temporal and spatial variabilities of atmospheric polychlorinated biphenyls (PCBs), organochlorine (OC) pesticides and polycyclic aromatic hydrocarbons (PAHs) in the Canadian Arctic: results from a decade of monitoring. Sci Total Environ.

[B20] Dewailly E, Ayotte P, Bruneau S, Laliberté C, Muir DCG, Norstrom RJ (1993). Inuit exposure to organochlorines through the aquatic food chain in Arctic Québec. Environ Health Perspect.

[B21] Blanchet C, Dewailly E, Ayotte P, Bruneau S, Receveur O, Holub BJ (2000). Contribution of selected traditional and market foods to the diet of Nunavik Inuit women. Can J Diet Pract Res.

[B22] Kinloch D, Kuhnlein H, Muir D (1992). Inuit foods and diet: a preliminary assessment of benefits and risks. Sci of Total Environ.

[B23] Gluer CC, Eastell R, Reid DM, Felsenberg D, Roux C, Barkmann R, Timm W, Blenk T, Armbrecht G, Stewart A, Clowes J, Thomasius FE, Kolta S (2004). Association of five quantitative ultrasound devices and bone densitometry with osteoporotic vertebral fractures in a population-based sample: the OPUS study. J Bone Miner Res.

[B24] Stewart A, Felsenberg D, Eastell R, Roux C, Glüer CC, Reid DM (2006). Relationship between risk factors and QUS in a European Population: The OPUS study. Bone.

[B25] Alfven T, Jarup L, Elinder CG (2002). Cadmium and lead in blood in relation to low bone mineral density and tubular proteinuria. Environ Health Perspect.

[B26] Wang H, Zhu G, Shi Y, Weng S, Jin T, Kong O, Nordberg GF (2003). Influence of environmental cadmium on forearm bone density. J Bone Miner Res.

[B27] Dequeker J, Ranstam J, Valsson B, Sigurgevisson B, Allander E (1991). The Mediterranean Osteoporosis (MEDOS) Study Questionnaire. Clin Rheumatol.

[B28] Phillips DL, Pirkle JL, Burse VW, Bernet JT, Henderson LO, Needham LL (1989). Chlorinated hydrocarbon levels in human serum: effects of fasting and feeding. Arch Environ Contam Toxicol.

[B29] Wallin E, Rylander L, Hagmar L (2004). Exposure to persistent organochlorine compounds through fish consumption and the incidence of osteoporotic fractures. Scand J Work Environ Health.

[B30] Bohannon AD, Cooper GS, Wolff MS, Meier DE (2000). Exposure to 1,1-dichloro-2,2-bis(p-chlorophenyl) ethylene (DDT) in relation to bone mineral density and rate of bone loss in menopausal women. Arch Environ Health.

[B31] Wallin E, Rylander L, Jonssson BA, Lundh T, Isaksson A, Hagmar L (2005). Exposure to CB-153 and *p*, *p*'-DDE and bone mineral density and bone metabolism markers in middle-aged and elderly men and women. Osteoporos Int.

[B32] Jamsa T, Viluksela M, Tuomisto JT, Tuomisto J, Tuukkanen J (2001). Effects of 2,3,7,8-tetrachlorodibenzo-p-dioxin on bone in two rat strains with different aryl hydrocarbon receptor structures. J Bone Miner Res.

[B33] Lind PM, Eriksen EF, Sahlin L, Edlund M, Orberg J (1999). Effects of the antiestrogenic environmental pollutant 3,3',4,4',5-pentachlorobiphenyl (PCB #126) in rat bone and uterus: diverging effects in ovariectomized and intact animals. Toxicol Appl Pharmacol.

[B34] Lind PM, Larsson S, Oxlund H, Hakansson H, Nyberg K, Eklund T, Orberg J (2000). Change of bone tissue composition and impaired bone strength in rats exposed to 3,3',4,4',5-pentachlorobiphenyl (PCB126). Toxicology.

[B35] Mazess RB, Mather W (1974). Bone mineral content of North Alaskan Eskimo. Am J Clin Nutr.

[B36] Mazess RB, Mather W (1975). Bone mineral content in Canadian Eskimo. Hum Biol.

[B37] Martin RB, Burr DB, Schaffler MB (1985). Effects of age and sex on the amount and distribution of mineral in eskimo tibia. Am J Phys Anthropol.

[B38] Filner JJ, Krohn KD, Lapidus JA, Becker TM (2002). Risk Factors for Osteoporosis in Alaska Native Women: A Cross-Sectional Survey. Alaska Med.

[B39] Gionet NJ, Godin G (1989). Self-reported exercise behavior of employees : A validity study. J Occup Med.

[B40] Frost ML, Blake GM, Fogelman I (2000). Can the WHO criteria for diagnosing osteoporosis be applied to calcaneal quantitative ultrasound?. Osteoporos Int.

[B41] Hans D, Dargent-Molina P, Schott AM, Sebert JL, Cormier C, Kotzki PO, Delmas PD, Pouilles JM, Breart G, Meunier PJ (1996). Ultrasonographic heel measurements to predict hip fracture in elderly women : The EPIDOS prospective study. Lancet.

[B42] Kishimoto H (2005). Change in the definition of osteoporosis especially on bone quality. Clin Calcium.

[B43] Thompson P, Taylor J, Fisher A, Oliver R (1998). Quantitative heel ultrasound in 3180 women between 45 and 75 years of age : compliance, normal ranges and relationship to fracture history. Osteoporos Int.

[B44] Rejnmark L, Jorgensen ME, Pedersen MB, Heickendorff L, Lauridsen AL, Mulvad G, Siggaard C, Skjoldborg H, Sorensen TB, Pedersen EB, Mosekilde L (2004). Vitamin D insufficiency in Greenlanders on a westernized fare : ethnic differences in calcitropic hormones between Greenlanders and Danes. Calcif Tissue Int.

